# Determination of Hydrophobic Dispersive Surface Free Energy of Activated Carbon Fibers Measured by Inverse Gas Chromatographic Technique

**DOI:** 10.3390/nano13061113

**Published:** 2023-03-20

**Authors:** Seul-Yi Lee, Yeong-Hun Kim, Roop L. Mahajan, Soo-Jin Park

**Affiliations:** 1 Department of Chemistry, Inha University, 100 Inharo, Incheon 22212, Republic of Korea; 2 Department of Mechanical Engineering and Institute for Critical Technology and Applied Science, Virginia Tech, Blacksburg, VA 24061, USA

**Keywords:** activated carbon fiber, hydrophobic surface component, surface free energy, inverse gas chromatography

## Abstract

Activated carbon fibers (ACFs) as one of the most important porous carbon materials are widely used in many applications that involve rapid adsorption and low-pressure loss, including air purification, water treatment, and electrochemical applications. For designing such fibers for the adsorption bed in gas and aqueous phases, in-depth comprehension of the surface components is crucial. However, achieving reliable values remains a major challenge due to the high adsorption affinity of ACFs. To overcome this problem, we propose a novel approach to determine London dispersive components ($\gamma_{S}^{L}$) of the surface free energy of ACFs by inverse gas chromatography (IGC) technique at an infinite dilution. Our data reveal the $\gamma_{S}^{L}$ values at 298 K for bare carbon fibers (CFs) and the ACFs to be 97 and 260–285 mJ·m^−2^, respectively, which lie in the regime of secondary bonding of physical adsorption. Our analysis indicates that these are impacted by micropores and defects on the carbon surfaces. Comparing the $\gamma_{S}^{L}$ obtained by the traditional Gray’s method, our method is concluded as the most accurate and reliable value for the hydrophobic dispersive surface component of porous carbonaceous materials. As such, it could serve as a valuable tool in designing interface engineering in adsorption-related applications.

## 1. Introduction

Since knowledge of surface-free energy is important in the design and assembly of materials, new strategies to harness these forces for different applications have caught the attention of the scientific and engineering communities. Consider, for example, the surface free energy ($\gamma_{S}$) of materials. It consists of two components: a London’s dispersive component ($\gamma_{S}^{L}$), resulting from the hydrophobic part, and a non-dispersive and polar component ($\gamma_{S}^{SP}$), which recently has been shown to be related to the concept of Lewis acid-base or electron acceptor-donor characteristics [1,2,3,4,5].

There are several methods to estimate surface physicochemical properties, such as the Brunauer-Emmet-Teller (BET) method, contact angle measurements, and inverse gas chromatography (IGC) technique. The BET method is widely used to determine the specific surface area (SSA) while contact angle measurement is used to evaluate polar component terms of surface free energy of a solid material. These methods, however, cannot provide all the general physicochemical information on a solid surface [6,7].

IGC technique is a versatile and robust surface energy measurement technique for investigating physicochemical surface properties of a solid material such as surface free energy, diffusion coefficients, phase transitions, and crystallinity. This method can determine the surface properties of solid organics, polymers, carbon materials, ceramics, etc., unaffected by morphologies of powders, pallets, films, and fibers. Furthermore, the IGC technique does not require controlled temperature and humidity thereby minimizing the effect of environmental conditions. In addition, the technique provides both the London dispersive component associated with SSA and the polar component-specific surface area that determines hydrophilicity at room temperature [8,9,10,11]. In implementing this technique, a solid is placed in an empty column, followed by the injection of known *n*-alkane probe solvents into the column as mobile phases. The adsorbent (a solid)-adsorbate (a probe) interaction is inferred from the retention time (defined as the time for a probe to elute through the column), which, in turn, provides a fundamental thermodynamic property of the interaction.

The widely used methods for determining London dispersive components using IGC were proposed by Dorris–Gray in 1980 [12] and Schultz et al., in 1987 [13]. Their calculations were based on the contribution of a series of *n*-alkanes to measure the free energy of adsorption, which due to the absence of acid-base interaction of the *n*-alkane probes leads to the determination of London dispersive component of surface free energy. A few studies that have reported on a comparison between Dorris–Gray and Schultz methods for determining $\gamma_{S}^{L}$ show contradictory results. Shi et al., estimated $\gamma_{S}^{L}$ following the two methods and concluded that the ratios $\gamma_{S}^{L}$ (Dorris–Gray)/$\gamma_{S}^{L}$ (Schultz) increased with increasing temperature [14]. Basivi et al., on the other hand, reported $\gamma_{S}^{L}$ (Schultz) to be higher than $\gamma_{S}^{L}$ (Dorris–Gray) [15].

In this paper, we propose a relatively unexplored way to determine the hydrophobic dispersive component of surface free energy of ACFs via IGC at infinite dilution and at room temperature. We demonstrate that compared to the traditional Dorris–Gray or Schultz method, this is a relatively simple and yet accurate method to determine surface energy profiles of highly porous carbonaceous materials. To demonstrate our approach, we have selected ACFs as exemplars since they are widely used in the fields involving rapid adsorption and low-pressure loss, including air purification, water treatment, and biomedical or electrochemical applications [16,17,18,19,20,21,22]. For instance, ACFs have been utilized as adsorbents for volatile organic compounds (VOCs) due to their extended surface area, fast adsorption–desorption rate, and high hydrophobic or non-polar adsorption capacity [18,23]. We note that London dispersive and specific components of carbon fibers (CFs), before and after modification by steam activation, have been determined experimentally to evaluate the adhesion between CFs and matrix using the IGC technique [24,25,26,27]. However, there is a lack of reliable experimental findings to relate the adsorption phenomenon with the London dispersive component ($\gamma_{S}^{L}$) of ACFs. To fill this gap, we present in this paper our experimentally determined values of $\gamma_{S}^{L}$ values at 298 K for three different ACFs. For comparison, these values were also determined for CFs.

## 2. Experimental

### 2.1. Materials

Pitch-based high strength carbon fibers were used in this work. The fibers were designated as 12 K (12,000 monofilaments), and 99 (surface non-treated and non-sized) by the supplier (Nippon oil Co. Ltd., Tokio, Japan). The pitch-based isotropic carbon fibers were heated up to 1173 K with a heating rate of 3 K·min^−1^, and then water-injected with 0.4 mL·min^−1^ for steam processing under 200 mL·min^−1^ flow of N_2_. The burn-off of different ACFs after the activation is listed in Table 1, which also lists the burn-off weight loss of carbon fibers, before and after the activation.

### 2.2. Textural Properties

The textural properties were investigated by N_2_/77 K volumetric adsorption analyzer (BELSORP-MAX; Microtrac BEL Inc., Osaka, Japan). Prior to the measurements, all samples were heated under 473 K for 12 h to remove the remaining organic species. The specific surface area was determined using Brunauer-Emmett-Teller (BET) equation.

### 2.3. IGC Measurements

Chromatographic measurements at infinite dilution were carried out with a GC-2014 gas chromatograph (Shimadzu Ltd., Kyoto, Japan) equipped with a flame ionization detector of extremely high sensitivity. The chromatograph was coupled with a Shimadzu, allowing analysis of the first moment of the elution peak to be made [8,11,25]. The CFs and the prepared ACFs were packed, respectively, into a 0.6 meter-length and a 0.01 meter-length of the 4.4 mm internal diameter of stainless-steel column. Prior to all measurements, degassing was carried out to remove any organic species at 373 K for 12 hours under helium gas flow. To achieve infinite dilution conditions, the n-alkanes probe vapor was collected by the constant rate syringe (CR700-20, Hamilton, OH, USA) from the sealed liquid bottle and successively five or more times) flushed out with air to dilute it. The amount of n-alkane probes injected corresponds to 0.01 mL, thus ensuring practically infinite dilution or zero surface coverage, with the adsorbed molecules being sufficiently far apart to neglect their mutual interaction. The flow rate of the helium carrier gas was 35 cm^3^·min^−1^, and the experimental column temperature varied from about 303 to 343 K. The amounts of carbon fibers and three types of activated carbon fibers were about 3.5 and 0.6 grams, respectively.

## 3. Results and Discussions

### 3.1. Inverse Gas Chromatography at Infinite Dilution

In this technique, the London’s dispersive component can be measured by Gray’s [12,28] or Park’s methods [8,29] using the adsorption of *n*-alkanes (non-polar probes) on a solid surface. In this work, we have determined and compared the values of $\gamma_{S}^{L}$ in highly microporous active carbon fibers, using both methods.

The classical and simple thermodynamic consideration of IGC at infinite dilution is given by [30]:

(1) $$-\Delta G_{A}=RT\;Ln\left(\frac{V_{n}\cdot P}{s\cdot g\cdot \Pi_{0}}\right)$$

where $-\Delta G_{A}$ is the adsorption (or desorption) free energy of one mole of solute from a reference state, and $V_{n}$, P, s, g, and $\Pi_{0}$ are, respectively, the net retention volume, and pressure of the solute, the specific surface area of a solid, weight of solid in the column, and bi-dimensional spreading pressure. It can be determined using

(2) $$-\Delta G_{A}=RT\cdot Ln\;V_{n}+C$$

where C is a constant depending on the chosen reference state, the temperature, the specific surface area, and weight of fibers in the column studied. Two reference states generally considered are those of Kemball and Rideal [31], where $P=1.013\times 10^{5}$ Pa and $\Pi_{0}=6.078\times 10^{-5}$ N·m^−1^; and De Boer [32], where $P=1.013\times 10^{5}$ Pa and $\Pi_{0}=3.38\times 10^{-4}$ N·m^−1^.

Two types of interaction are assumed in Equation (2). These are:

(3) $$=\left(-\Delta G_{A}^{L}\right)+\left(-\Delta G_{A}^{SP}\right)$$

where the superscript L and SP refer to the London’s dispersive or non-polar, and the specific or polar interaction, respectively.

The net retention volume can be determined in the chromatographic experiment as:

(4) $$V_{n}=j\cdot D\cdot \left(t_{R}-t_{0}\right)$$

where $t_{R}$ is the retention time of the given probe, $t_{0}$ the zero-retention reference time measured with a practically non-adsorbing probe such as methane, D the flow rate, and j a correction factor taking into account gas compressibility.

The London dispersive component ($\gamma_{S}^{L}$) of the solid surface energy using Gray’s method [12,28] can be calculated as:

(5) $$\gamma_{S}^{L}=\frac{{\left(-\Delta G_{CH_{2}}\right)}^{2}}{4N_{A}^{2}a_{CH_{2}}^{2}\gamma_{CH_{2}}}$$

where $\Delta G_{CH_{2}}$, the incremental free adsorption (or desorption) energy of methylene group ($CH_{2}$), is given by:

(6) $$-\Delta G_{CH_{2}}=RT\cdot Ln\left(\frac{V_{n+1}\left(C_{n+1}H_{2n+4}\right)}{V_{n}\left(C_{n}H_{2n+2}\right)}\right)$$

Here, $N_{A}$ is the Avogadro constant (6.022 × 10^23^ mol^−1^), $a_{CH_{2}}^{2}$ is the surface of area of $CH_{2}$ assumed by Gray et al., as 6 ${Å}^{2}$, and $\gamma_{CH_{2}}$ is the surface free energy of a $CH_{2}$ group given by:

(7) $$\gamma_{CH_{2}}=35.6-0.058\left(t-20\right)\;\mathrm{in}\;\mathrm{mJ}\cdot m^{-2}.$$

t being the temperature in Celsius. Substituting the calculated value of $N_{A}\cdot a_{CH_{2}}^{2}=$ 36,132 m^2^·mol^−1^, Equation (5) reduces to:

(8) $$\gamma_{S}^{L}=\frac{1}{4\gamma_{CH_{2}}}{\left(\frac{RT\cdot Ln\left(\frac{V_{n+1}}{V_{n}}\right)}{36132}\right)}^{2}\;\mathrm{in}\;[\mathrm{mJ}\cdot m^{-2}]$$

Following Park et al. [8,29], we can rewrite the London dispersive component ($\gamma_{S}^{L}$) of the solid surface energy ($\gamma_{S}$) as the arithmetic mean for the two parameters of $\gamma_{S}^{L}$ and $\gamma_{CH_{2}}^{L}$ [29] as:

(9) $$\gamma_{S}^{L}=\frac{-\Delta G_{A}\left(CH_{2}\right)}{N_{A}\cdot a_{CH_{2}}^{2}}\;\mathrm{in}\;[\mathrm{mJ}\cdot m^{-2}]$$

With $N_{A}\cdot a_{CH_{2}}^{2}=$ 36,132 m^2^·mol^−1^, as before, Equation (9) reduces to as:

(10) $$\gamma_{S}^{L}=\frac{-\Delta G_{A}\left(CH_{2}\right)}{36132}\;\mathrm{in}\;[\mathrm{mJ}\cdot m^{-2}]$$

### 3.2. London’s Dispersive Surface Free Energy

**Figure 1:**
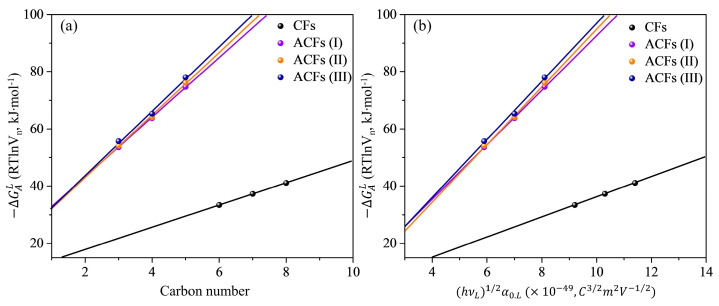
The concept for determining London’s dispersive component of surface free energy by (**a**) Gray’s and (**b**) Park’s methods. (Standard error values of slopes are CFs, ACFs (I), ACFs (II), and ACFs (III) for (**a**) 0.0288, 0.2021, 0.5774, and 0.8949 and (**b**) 0.0262, 0.1837, 0.5249, and 0.9945, respectively).

The next important parameter to be determined is $-\Delta G_{A}^{L}\left(CH_{2}\right)$. According to equation (6), this can be easily determined by the value of slope of the linearly fitted Gibbs free energy of methylene group at 298 K. The so-obtained values $-\Delta G_{A}^{L}\left(CH_{2}\right)$ at 298 K, calculated by both Gray’s and Park’s methods, are shown in Figure 1 and listed in Table 1.

**Figure 2:**
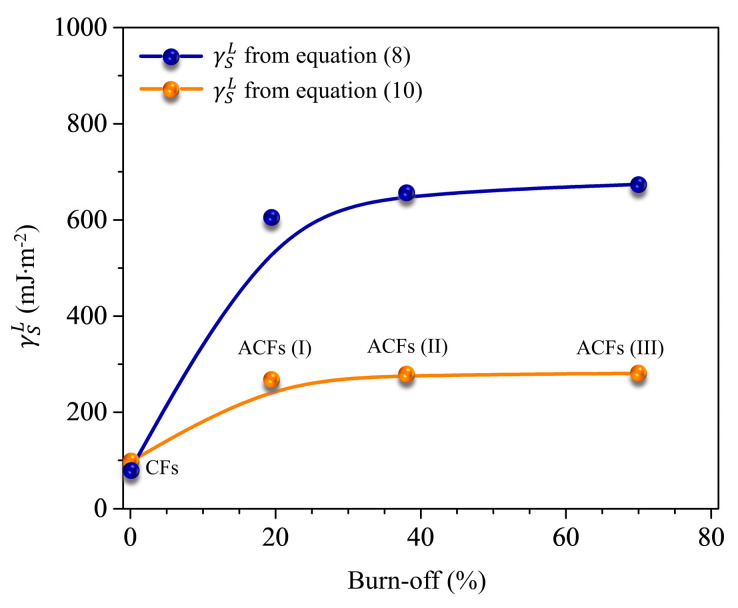
Comparison of the London dispersive surface free energy determined from Gray’s and Park’s methods.

With $-\Delta G_{A}^{L}\left(CH_{2}\right)$ known for various *n*-alkanes (C3–C8), which are only able to exchange dispersive interactions, we can now easily determine London’s dispersive surface free energy ($\gamma_{S}^{L}$) of the solid surface determined by Park’s method, Equation (10). For Gray’s method (Equation (8), it was found additional computation of the term $\frac{RT\cdot Ln\left(\frac{V_{n+1}}{V_{n}}\right)}{36,132}$ is needed. The obtained values of $\gamma_{S}^{L}$ determined from the Gibbs free energy ($-\Delta G_{A}^{L}\left(CH_{2}\right)$) at 298 K are shown in Figure 2.

As previously reported by several researchers [24,25,26,27,33], it is now generally accepted that the $\gamma_{S}^{L}$ of an ACF sample either determined from Gray’s method, Equation (8), or Park’s method, Equation (10), can be taken as a measure of its surface free energy. However, as Figure 2 shows that the values of $\gamma_{S}^{L}$ calculated from Gray’s method are much greater than those determined using Park’s method. They are in the typical primary region (e.g., metal–metal bonds of mercury and tin are 475 and 526 mJ·m^−2^, respectively) [34]. Using our approach, they are ~300 mJ·m^−2^ and are consistent with the regime dominated by physical adsorption, which seems a more reasonable interpretation for highly porous and hydrophobic materials.

As mentioned above, the degree of London’s dispersive surface free energy of highly porous and hydrophobic materials plays a major role in the evaluation of its hydrophobicity. As expected, the values of specific surface area (S_BET_) for the ACFs increased with increase in the degree of burn-off (Table 2). However, it was confirmed that the obtained $\gamma_{S}^{L}$ values remain nearly constant as a function of the degree of burn-off, see Figure 2, suggesting that the values of $\gamma_{S}^{L}$ at infinite dilution condition are not proportional to the S_BET_ or the degree of burn-off (%)—a desirable result for practical applications. In other words, the $\gamma_{S}^{L}$ values determined from the simple IGC technique at infinite dilution indicates the representative surface free energy of the most prominent active sites on the ACF surfaces at room temperature.

**Figure 3:**
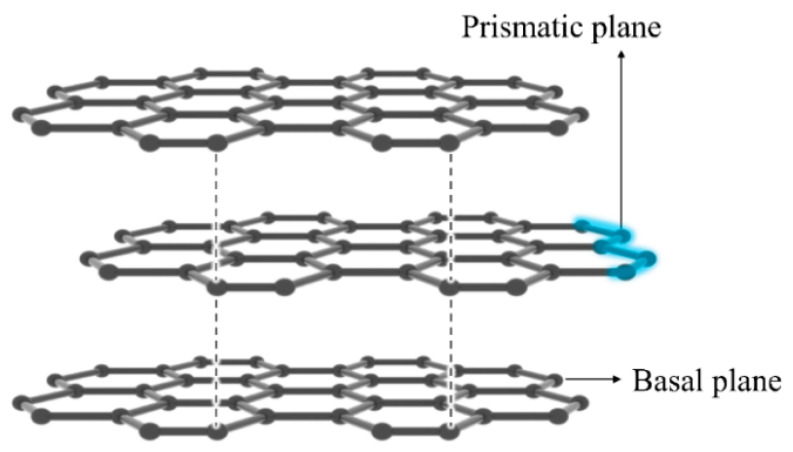
Structure of graphite.

Finally, we note that in graphite, the surface free energy at room temperature between basal and prismatic planes varies anywhere between three to five times [35,36,37], see Figure 3. This might be due to the prismatic sites on the prismatic plane that give rise to increased surface free energy. By extension, micropores and/or defects at the prismatic planes in porous and hydrophobic carbonaceous materials may act as a primary factor to determine the surface free energy in this system.

## 4. Conclusions

In this paper, we have proposed a new approach to determine the London dispersive component ($\gamma_{S}^{L}$) of surface free energy using a variation of London dispersive component of n-alkanes ($\gamma_{CH_{2}}^{L}$) via inverse gas chromatography at an infinite dilution. The values of $\gamma_{S}^{L}$ at 298 K for bare carbon fibers (CFs) and the ACFs (ACFs (I), (II), and (III)) were determined to be 97, 266, 277, and 281 mJ·m^−2^, respectively. For the ACFs, the values are in the regime of secondary bonding, i.e., physical adsorption and intermolecular long-range interaction. In comparison, the $\gamma_{S}^{L}$ values for the ACFs determined from Gray’s method are in the range of 604–674 mJ·m^−2^. Considering that typical metal–metal primary bond energetics of mercury are ~475 mJ·m^−2^, accuracy of the $\gamma_{S}^{L}$ value obtained by Gray’s method is questionable [38]. The results also point to an important difference between CFs and AFCs. The burn-off treatments on CFs lead to an increase of the specific surface area or their hydrophobicity. However, the results obtained from IGC at infinite dilution indicate that the hydrophobic dispersive surface free energy of porous materials is greatly related to the specific surface area only in the domain where the intermolecular adsorption process is considered adsorbate–adsorbent. We can therefore infer that our suggested method would be more accurate and reliable in determining the $\gamma_{S}^{L}$ for porous materials.

## Figures and Tables

**Table 1:** Comparison of the London dispersive component from Gibbs free energy, $-\Delta G_{A}^{L}\left(CH_{2}\right)$, of the samples at 298 K calculated by Gray’s and Park’s methods.

Samples	$-\Delta G_{A}^{L}\left(CH_{2}\right)$ at 298 K (kJ·mol^−1^)	
	Gray	Park
CFs	3.9	3.5
ACFs (I)	10.6	9.6
ACFs (II)	11.0	10.0
ACFs (III)	11.2	10.1

**Table 2:** Burn-off and specific surface area of samples.

Samples	^a^ Burn-off (wt.%)	^b^ S_BET_ (m^2^·g^−1^)
CFs	0	-
ACFs (I)	19.5	340
ACFs (II)	38.0	500
ACFs (III)	70.0	1630

^a^ Burn-off $=\left(m_{initial}-m_{post}\right)\times 100/m_{initial}$; ^b^ Specific surface area computed using BET equation at a relative pressure range of 0.001–0.01.

## Data Availability

Not applicable.
